# Supplementary material for: Expanding the Pathogen Panel in Wastewater Epidemiology to Influenza and Norovirus
Source: Viruses. 2023 Jan 17;15(2):263. doi: 10.3390/v15020263 (PMC9966704; doi:10.3390/v15020263)
Supplement: Supplementary file 1 [file viruses-15-00263-s001.zip › viruses-2092381-supplementary.pdf]

Table S1 Primers and Probes for IAV, IBV, NoV GI and NoV GII detection via RT-dPCR

**Primer/probe target**	**Description**	**Sequence (5′–3′)**
Influenza A	InfA For1	CAA GAC CAA TCY TGT CAC CTC TGA C
	InfA For2	CAA GAC CAA TYC TGT CAC CTY TGA C
	InfA Rev1	GCA TTY TGG ACA AAV CGT CTA CG
	InfA Rev2	GCA TTT TGG ATA AAG CGT CTA CG
	InfA Probe	FAM/TGC AGT CCT CGC TCA CTG GGC ACG/BHQ-1
Influenza B	InfB For	TCC TCA AYT CAC TCT TCG AGC G
	InfB Rev	CGG TGC TCT TGA CCA AAT TGG
	InfB Probe	HEX/CCA ATT CGA GCA GCT GAA ACT GCG GTG/BHQ-1
Norovirus GI	LZIF	GGA GAT CGC RAT CTC CTG CCC GA
	LZIR-A	CTC YGG TAC CAG CTG GCC
	LZIR-B	CCT CYG GHA CCA GCT GAC C
	LZIP	HEX/CGT CCT TAG ACG CCA TCA TCA TTT AC/BHQ-1
Norovirus GII	LZIIF-A	GTG GGA TGG ACT TTT ACG TGC CAA G
	LZIIF-B	GGT GGM ATG GAT TTT TAC GTG CCC AG
	LZIIR	CGT CAY TCG ACG CCA TCT TCA TTC AC
	LZIIP	FAM/AGC CAG ATT GCG ATC GCC/BHQ-1

Table S2 Linearised DNA positive-control sequences after in vitro amplification

NoV GI	5’_GGAGCAGACAAGCCCGTCAGGGCGCGTCAGCGGGTGTTGGCGGGTGTCGGGGCTGGCTTA<br>ACTATGCGGCATCAGAGCAGATTGTACTGAGAGAAAGGCAATTGGGTACCGAGCTCGCGGCCG<br>CAAGCGGACA***GGAGATCGCAATCTCCTGCCCGAATTCGTAAATGATGATGGCGTCTAAGGAC***<br>***GCCCCAACATCCCCTGATGGCGCCAGTGGCGCCGGCCAGCTGGTACCGGAGGC***TAATAACCTG<br>CTTTTGCTCGCTTGGATCCGAATTCAAAGGTGAAATTGTTATCCGCTCACAATTCCACACAACAT<br>ACGAGCCGGAAGCATAAAGTGTAAAGCCTG_3’
NoV GII	5’_GGAGCAGACAAGCCCGTCAGGGCGCGTCAGCGGGTGTTGGCGGGTGTCGGGGCTGGCTTA<br>ACTATGCGGCATCAGAGCAGATTGTACTGAGAGAAAGGCAATTGGGTACCGAGCTCGCGGCCG<br>CAAGCAGAAG***GTGGGATGGACTTTTACGTGCCAAGGCAGGAACCCATGTTCAGGTGGATGAG***<br>***GTTTTCTGACTTGAGCACGTGGGAGGGCGATCGCAATCTGGCTCCCAATTTTGTGAATGAAGA***<br>***TGGCGTCGAATGAGC***CGCTCACCTGCTTTTGCTCGCTTGGATCCGAATTCAAAGGTGAAATTGT<br>TATCCGCTCACAATTCCACACAACATACGAGCCGGAAGCATAAAGTGTAAAGCCTG_3’
IAV	5’_GGAGCAGACAAGCCCGTCAGGGCGCGTCAGCGGGTGTTGGCGGGTGTCGGGGCTGGCTTA<br>ACTATGCGGCATCAGAGCAGATTGTACTGAGAGAAAGGCAATTGGGTACCGAGCTCGCGGCCG<br>CAAGCAAAGA***CAAGACCAATCCTGTCACCTCTGACTAAGGGGATTTTAGGGTTTGTGTTCACG***<br>***CTCACCGTGCCCAGTGAGCGAGGACTGCAGCGTAGACGCTTTGTCCAAAATGC***CCTAAACCTG<br>CTTTTGCTCGCTTGGATCCGAATTCAAAGGTGAAATTGTTATCCGCTCACAATTCCACACAACAT<br>ACGAGCCGGAAGCATAAAGTGTAAAGCCTG_3’
IBV	5’_GGAGCAGACAAGCCCGTCAGGGCGCGTCAGCGGGTGTTGGCGGGTGTCGGGGCTGGCTTA<br>ACTATGCGGCATCAGAGCAGATTGTACTGAGAGAAAGGCAATTGGGTACCGAGCTCGCGGCCG<br>CAAGCTCGGA***TCCTCAATTCACTCTTCGAGCGTCTTAATGAAGGACATTCAAAGCCAATTCGAG***<br>***CAGCTGAAACTGCGGTGGGAGTCTTATCCCAATTTGGTCAAGAGCACCG***ACTATACCTGCTTTT<br>GCTCGCTTGGATCCGAATTCAAAGGTGAAATTGTTATCCGCTCACAATTCCACACAACATACGA<br>GCCGGAAGCATAAAGTGTAAAGCCTG_3’
Pex-plasmid-residue, overhang, ***selective primer-region,*** ***target-sequence with probe-region***	

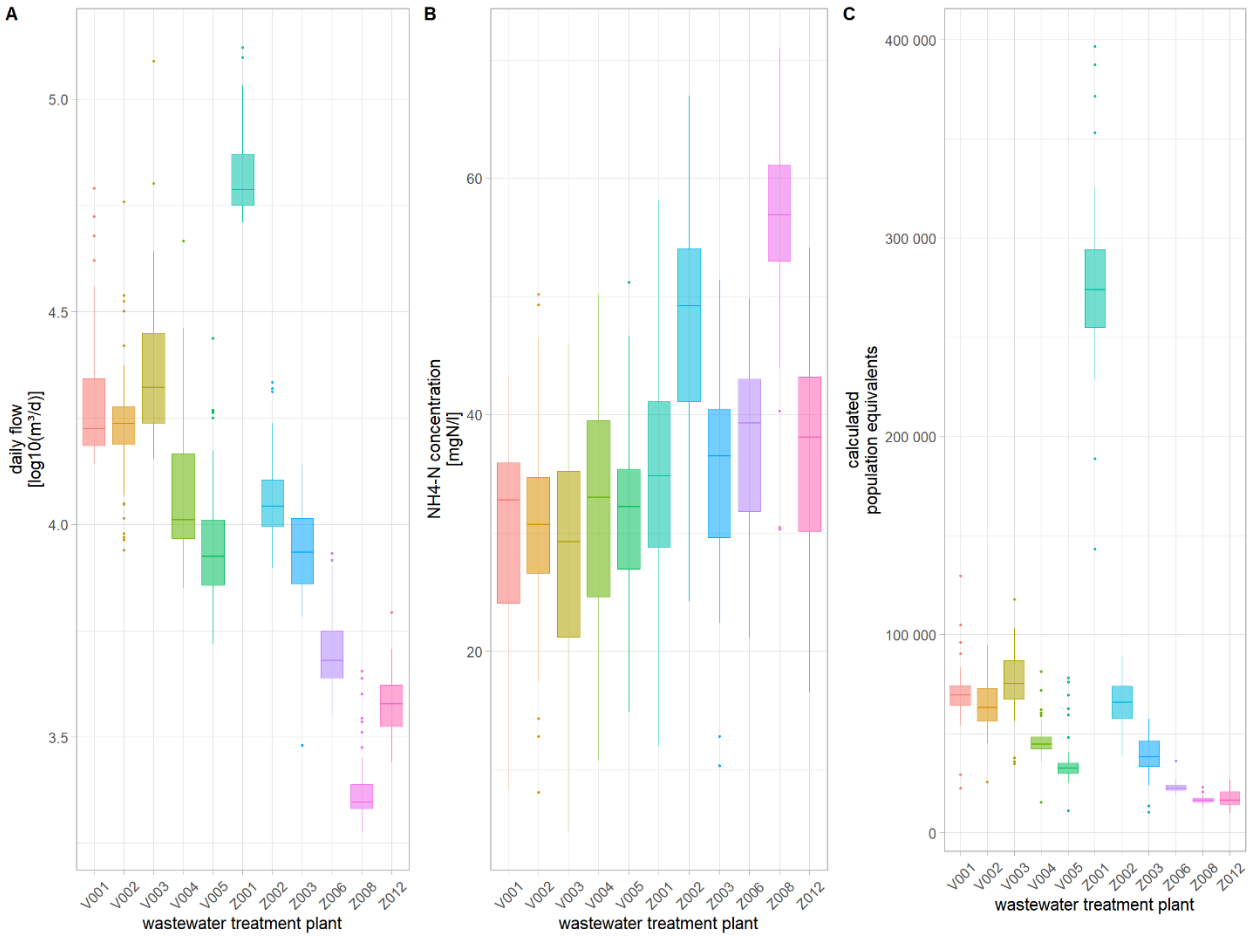

Figure A1 Flow rate, NH_4-N concentration and calculated population size according to NH_4-N normalization.

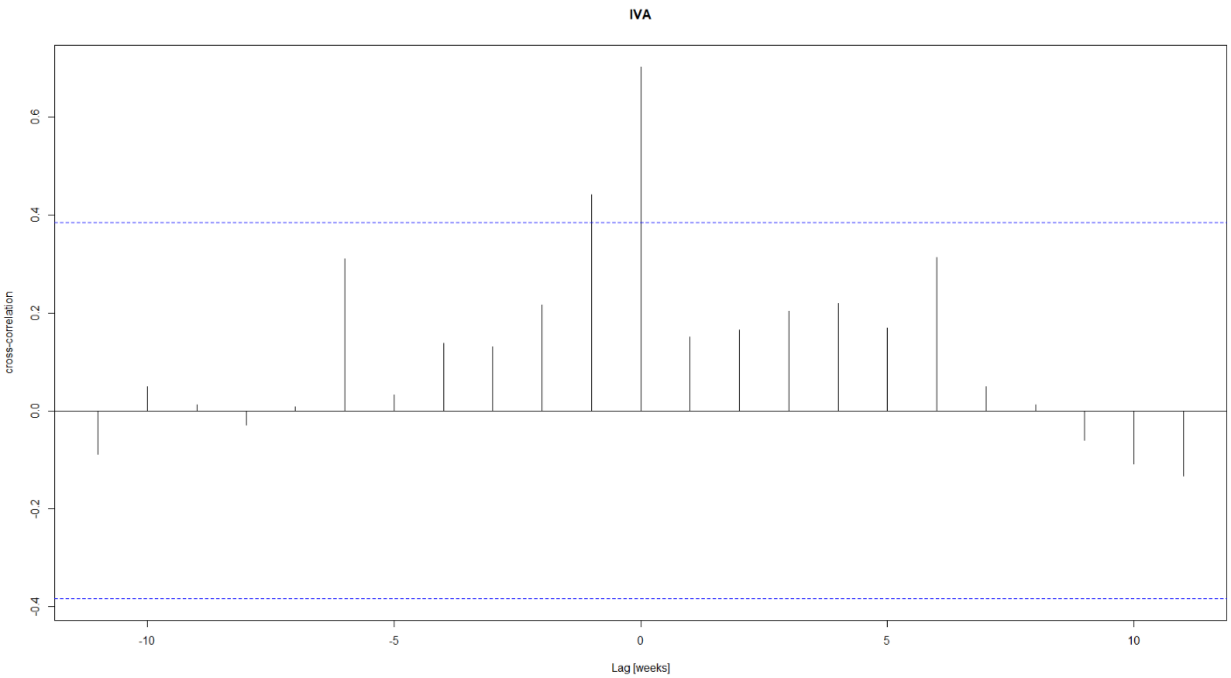

Figure A2 Cross correlation analysis for IVA and inpatients infected with influenza virus

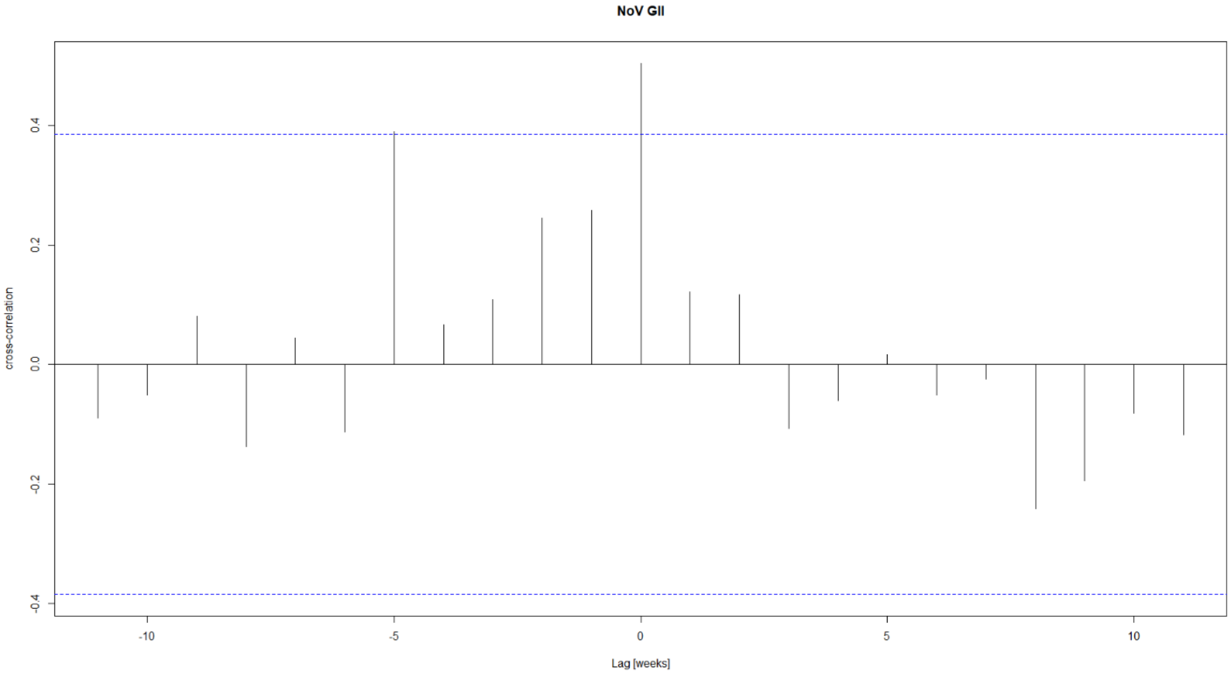

Figure A3 Cross correlation analysis for NoV GII and inpatients infected with NoVs.
